# Personalized Health Care and Public Health in the Digital Age

**DOI:** 10.3389/fdgth.2021.595704

**Published:** 2021-03-30

**Authors:** Oliver Y. Chén, Bryn Roberts

**Affiliations:** ^1^Department of Engineering, University of Oxford, Oxford, United Kingdom; ^2^Division of Biosciences, University College London, London, United Kingdom; ^3^Roche Pharmaceutical Research and Early Development, Roche Innovation Center, Basel, Switzerland

**Keywords:** digital health, personalized healthcare, public health, AI in healthcare, digital biomarkers, FAIR data, telemedicine, mHealth

## Introduction

Personalized health care and public health are essential to one's well-being and societal welfare. The former focuses on symptoms and disease progression at the individual level, whereas the latter looks at health issues at the population level (from a group of patients to everyone on the planet). Recent years have seen a digital revolution in personalized health care and public health (1–4). As such, the World Health Organization regards mobile health (mHealth) as a vital resource for health services delivery and public health (5) and urges its Member States to prioritize the development and use of digital technologies in health to promote Universal Health Coverage and advance the Sustainable Development Goals (6).

Here, we discuss how digital health contributes to personalized health care and public health and how it may evolve. Specifically, we present its roles, challenges, and potential future.

## The Roles of Digital Health in Personalized Health Care and Public Health

### Self-Monitoring Health and Disease

Traditional health assessments require in-clinic visits, with bio-signals measured by professionals using specialized devices. To practice these frequently in a broad population is, however, inconvenient and expensive.

**Figure 1:**
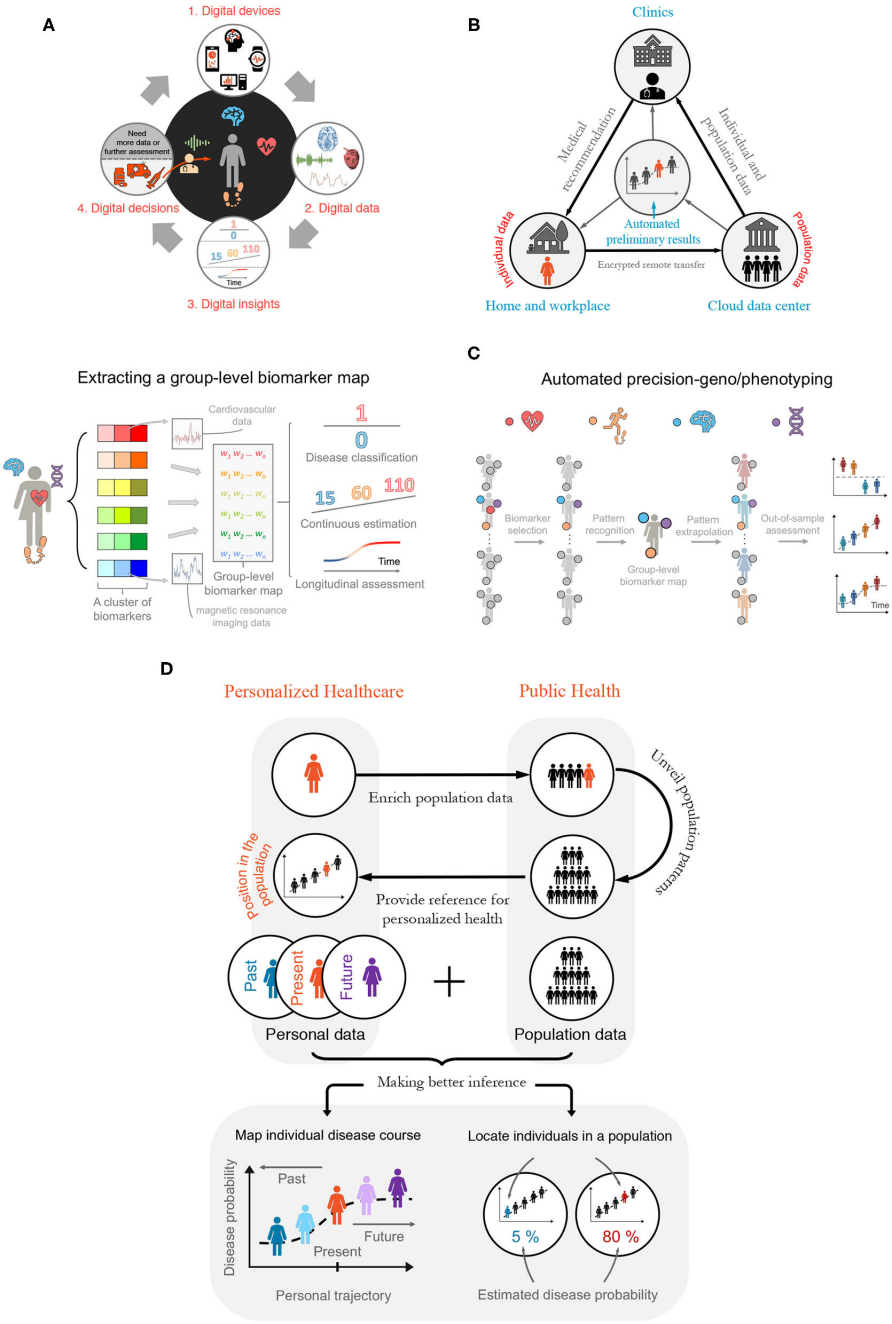
Building a Digitalized Ecosystem. **(A)** Digitalization cycle—bridging digital health, data, and digitally enabled decision-making. From 1 to 2: Phenotypic information is recorded by, and made available on, digital devices. From 2 to 4 *via* 3: Advanced modeling and analyses are applied on digital data to guide health-care decisions by physicians, such as recommending potential treatments (see bottom of item 4) and suggesting conducting further diagnostic tests or analyses (from 4 to 1). **(B)** Semi-automated disease diagnosis and monitoring. Individual data are de-identified, encrypted, and transferred remotely *via* the cloud to secured data centers—where population-level disease analyses are performed. The individual data are then analyzed and compared with the population features to generate an automated preliminary diagnosis report. Should red flags be raised, the report is passed on to a medical professional. The medical professional looks at both the report and the individual-specific data to offer medical recommendations or to arrange for an on-site visit or remote consultation (e.g., telemedicine), depending on which further tests are anticipated. This process continues between an individual's periodic health checks to ensure earliest possible detection of symptoms and to compensate for long consultation intervals when regular assessment is too expensive or inconvenient. **(C)** Automated deep-geno/phenotyping. The digital health applications facilitate ensemble-learning, where multivariate genotypic and phenotypic (e.g., digital biomarker, imaging, and molecular diagnostics) data are combined to provide rich subject-specific and group-level information, which are used to position individuals within a relevant population or to predict outcomes for individuals, based on population norms. **(D)** The marriage between personalized health care and public health. There is a powerful interdependent and synergistic relationship between personalized health care and public health in the digital age. Top: When shared, personal digital health data bring rich heterogeneous information into the population data pool. In return, features identified in large-scale public health data will provide a reference for individuals, positioning them relative to comparable cohorts in the population, based on age, sex, ethnicity, *etc*. Bottom: Integrating individual and population data. Data collected longitudinally over months and years enable risk estimation and disease forecasts, such as estimating the likelihood of disease progression or quantifying response to different treatments.

Digital devices are mobile (or wearable), with fast data transmission done wirelessly. This enables remote and non-invasive health assessments and encourages users to self-care for their health (Figure 1A) (7, 8). Frequent assessments facilitate early disease discovery and longitudinal monitoring (7). Self-monitoring benefits those whose diseases may progress between hospital visits; the relatively affordable price (compared to hospital costs) makes health service accessible to many without in-hospital accesses (9). Self-monitoring does not confront in-clinic assessments. The former is as-of-yet not as accurate or comprehensive, but it provides complementary services and is more ecological to capture fluctuating symptoms.

### Semi-Automated Assessment and Diagnosis

Semi-automated health service alleviates a shortage in medical data, personnel, and equipment in several ways. First, it automates large-scale data collection. Next, automated data de-identification, encryption, and transfer (10) allow comparing personal data with population counterparts (11). Third, automation supports early disease detection, intervention, and treatment (12–15). For example, algorithms generate alerts suggesting medical consultation or delivering reports, with consent, to clinicians to assign interventions (16, 17) (see Figure 1B and telemedicine below).

Naturally, one would ask, will digitalization and automation of health service result in job losses, for example, rendering health-care personnel, nurses, and physicians redundant? Likely not. First, such posts are in shortage (18). Although much of monitoring and assessment can be done digitally, the majority of high-value medical interventions cannot be automated at present or in the near future. The expansion of overall coverage, therefore, results in an increasing need for service to deal with the tasks that cannot be automated. Second, digital transformation and revolution (see section Long-Term Outlooks) grow the labor market by creating new jobs, such as data labeling, medical testing and analysis, and remote medical service (e.g., telemedicine). It also creates and expands domain-specific positions, such as programming, posts requiring interdisciplinary knowledge, and educational jobs training the next-generation digital-health workforce (19).

The diagnosis made by physicians are not always correct (20, 21). Automated health services also make mistakes. Some machine-based predictions, however, have begun to outperform specialists in mammographic screening (22), certain cancer diagnosis (4) [e.g., urothelial carcinoma (23)], and retinal analyses (24). They also contribute to predicting cardiovascular disease (17), Lyme disease (11), neurodegenerative diseases (15), and treatment response (25). Yet, algorithmic caution and human safeguard, such as introducing a two-layer (digital plus human) verification system, are high-priority (Figure 1B). Although one can fine-tune parameters to balance false positives and negatives, better methods and higher-quality and larger data aggregation are needed to improve model performance.

### Connecting and Facilitating Personalized Health Care and Public Health

Digital health connects and facilitates personalized health care and public health (Figure 1D). First, accumulating individual data expands the population data repertoire. The convenience of digital data collection enables the inclusion of representative samples with balanced group sizes. This is helpful for distinguishing diseases with subcategories. Suppose a dataset has dominating Type 1 diabetes patients. The extracted knowledge would reflect Type 1 diabetes-specific features and the Type 2 diabetes-specific features may be overlooked or treated as noise. Currently, the majority of phenotypic health data are generated in the clinic from patients with relatively advanced diseases, where symptoms are clearly present and diagnostic procedures are being or have been performed. There is a lack of comparable data at scale from the healthy population and those in the very early stages of disease development. With representative samples and balanced group sizes, digital data support building more robust predictive models.

Second, information learned from population data improves personalized treatment. Digital devices are sensitive, objective, and collect data semi-continuously; they facilitate deep phenotyping (26, 27), finer disease categorization, and timely treatment (Figure 1C). If a patient displays features similar to those of a population, one can assign treatments shown safety and efficacy in the population. Comparing subject-specific treatment responses, side-effects, and symptoms with population counterparts, one can refine personalized treatments (28–30).

Integrating population and individual data yields better longitudinal inference. The longitudinal analysis helps painting health trajectories and forecasting future events. Frequently comparing individual information with personal and population history helps making better inference (Figure 1D).

## The Future of Digital Health

The digital age is still at its dawn. Here, we first examine, exemplarily, digital health's short-term focus in managing pandemics. We then discuss the mid-term challenges, for which one can actively prepare and make constructive improvements. Finally, we speculate, in an informed way, its long-term perspectives.

### Short-Term Focus: Digital Health During the Coronavirus Pandemic

Testing is chief during pandemics. It identifies and isolates infected individuals (especially the asymptomatic ones), facilitates timely treatment, and provides governments with feedback on “social distancing” measures. Unfortunately, by January 2020, manufacturers could only produce 100,000 COVID-19 testing kits *per* day when three million were in quarantine around Wuhan (31). From February to April 2020, the United States performed about 4.5 million tests (32), compared to suggested *daily* tests of 5–20 million (33).

Digital devices support testing, track-and-trace, and marking high-risk populations and areas (but see privacy concerns in section Mid-Term Challenges and Potential Solutions). Tencent and Alibaba provided users in China with a “health code”: one with a green, yellow, or red code, respectively, can travel, should stay home, or is a confirmed case and quarantined (34). Several European countries introduced smartphone apps to identify and inform individuals at risk (35). Real-time in-hospital monitoring enables staff to assist more people or focus on critical patients (36) (Figure 1C).

### Mid-Term Challenges and Potential Solutions

Digital health enables frequent, remote, and semi-automated health services. Yet, it is not without challenges.

Immediate concerns are data privacy (37) and quality (38). To secure data collection, transfer, storage, and analyses, we need improved government policies (39), company regulations (40), and computational and storage technology (1). To warrant the quality, we need quality controls, enhanced algorithms, and rigorous training and test.

Most digital solutions are domain-specific: for each disease, a new model needs to be developed (2, 4, 11, 15, 17). Yet, for those wishing to detect disease early without knowing which disease(s) may develop, a battery of bio-signals needs to be analyzed on a single device (Figure 1C). The fragmentation of health care and a lack of global data standards, however, make combining datasets and algorithms difficult (29, 41–43). Beginnings, however, are made, for example, by introducing a digital ward (36), the findability, accessibility, interoperability, and reusability (FAIR) data principles, and ensemble learning (41), to provide transparent, reproducible, and reusable services (44) and to better connect devices, algorithms, and datasets.

Although the elderly benefit from digital health (7, 45), they may not accept it (46, 47). It is partially because it is a recent concept, and partially because devices are difficult to use. Better introduction and developing easy-to-use devices may make digital health more accessible to them.

Finally, the talent challenge—the rarity of professionals with interdisciplinary knowledge. At present, a doctor may need to consult with data scientists to interpret the output of automated algorithms. A deep-learning engineer may need domain experts to explain the clinical concepts and medical situation. Disciplinary experts together with multi-disciplinary teams are essential in research and development today and will continue to be an integral, indispensable part in the future. A new generation of professionals with interdisciplinary training in life science, medicine, public health, and the computational domains, however, will be in high demand in the coming years. Positioned at the nexus of disciplines, they will be the integrators in complex teams, bridging multiplex interactions, multi-domain operations, and disciplinary experts.

### Long-Term Outlooks

Digital health will continue to support personalized health care and public health. Yet, it is difficult to fully predict its long-term outlooks. We can only speculate, in an informed way, through what we see today.

#### The Advent of Digital Health Ecosystems?

We may see an expedited digital health revolution, thanks to the fourth industrial revolution (Industry 4.0) supported by 5G and the Internet of Things (48). Leveraging real-time analytics, machine learning, commodity sensors, and embedded systems (49), Industry 4.0 connects patients, machines, and medical personnel (50). Such connectivity allows multivariate *big data* collection from heterogeneous populations. The frequent, longitudinal analyses on big data improve patient identification, severity estimation, and progression monitoring (Figure 1C).

Future may witness digital therapy. The augmented and virtual-reality exergaming, brain-machine interface (51) may treat substance-use disorder (52) and children with attention deficit hyperactivity disorder (53), among others (51, 54–57). For broad adoption and reimbursement, however, these interventions need to demonstrate safety and health-economic benefits (58).

Eventually, digital health ecosystems and digital clouds may emerge (Figure 1B). With more data and improved algorithms, the ecosystems become ever-smarter (59) (Figure 1D). They automate labor-intensive tasks and reshape the roles humans play (e.g., humans will focus on developing and maintaining digital devices and systems or deploying interventions when results are modest and sensitive decisions are needed).

Timing is important: the ecosystems are multi-dimensional (e.g., scientific, technical, social, political, and economic); a stagnation in one dimension may delay the whole. Equally important is making applications that are powerful and easy-to-use (“killer applications”). Unfortunately, timing and “killer applications” are oftentimes obvious retrospectively. New ideas and enthusiasm, despite failures, will bring us progressively closer to success.

## Discussion

Central to digital health is ethics (60, 61). To ensure digital recommendations do not disadvantage particular groups, we need representative samples and reproducible models. Representative samples span characteristics (e.g., gender, ethnicity, age, and devices) of the target population. Reproducible models are generalizable to out-of-sample individuals (Figure 1C). Additional quality control can ascertain the quality and ethical standards of different artificial intelligence algorithms (62). Future devices need to demonstrate robust services in a live, potentially noisy environment (63, 64). They also need to incorporate user feedback, such as emotions and verbal inputs, to improve personalized medical judgements (65–70).

Brain studies inspired early deep-learning models (71, 72). The layers in the convolutional neural networks in vision studies are reminiscent of the hierarchy in the visual cortex (73). Future may see more models resembling biological systems. Some may produce human-like characteristics. But we need to evaluate whether the similarities are generalizable to other models, higher functions, and organs, and to ensure the machine follows ethics and autonomy (74, 75).

We have covered the responsibility and functionality aspects of the digital health above. Digital health ecosystems, however, need to also consider explainability and interpretability (76, 77). Many state-of-the-art (deep learning) models at present, however, are potentially *explainable* [one may not understand the model after it is fit but can use another model (or test) to make sense of it (76, 78)] but not *interpretable* [one cannot understand how the model produces outcomes causally (76)]. Additionally, between interpretability and performance, there is typically an inverse relationship (77). For security or privacy, companies or governments may even obfuscate models.

Digital health benefits all of us, current or future patients desiring early diagnosis and timely treatment. But there are sizeable challenges we must overcome. Combining efforts from academia, industry, hospitals, non-profit, and government, we are hopeful to make digital health services effective and affordable.

## Author Contributions

Both authors conceptualized the study and wrote the paper together.

## Conflict of Interest

BR is employed by F. Hoffmann-La Roche; OC has consulted for F. Hoffmann-La Roche.
